# Supplementary material for: Integrated Genomic and Transcriptomic Elucidation of Flowering in Garlic
Source: Int J Mol Sci. 2022 Nov 10;23(22):13876. doi: 10.3390/ijms232213876 (PMC9698152; doi:10.3390/ijms232213876)
Supplement: Supplementary file 1 [file ijms-23-13876-s001.zip › ijms-1955352-supplementary Table S2 and Figures S1 and S2.pdf]

```
                  10         20         30         40         50         60
         ....|....| ....|....| ....|....| ....|....| ....|....| ....|....|
TFL1.1   --------MA RATDTLVVGR VIGEVLDNFS PSVKIVVTYN SNKLVCSGHE FFPSIVAPKP
TFL1.2   --------MA RATDTLVVGR VIGEVLDNFS PSVKIVVTYN SNKLVCSGHE FFPSIVAPKP
TFL1.3   --------MA RATDTLVVGR VIGEVLDNFS PSVKIVVTYN SNKLVCSGHE FFPSIVAPKP
TFL1.5   ------MARA RATGTLAVGR VIGEVLENFN PSAKIVVTYN TNKLVCSGHE FFPSVVAPKP
TFL1.4   NSPIKLISMA RATRTLAVGR VIGEVLENFN PSVKIVVTYN SNKLVCSGHE FFPSVVASKP
CEN      --------MS RVMDPLSVGR VIGDVMDAFD PSVKMVVSYG T-KQVYNGHE LMPAVVCSKP

                  70         80         90        100        110        120
         ....|....| ....|....| ....|....| ....|....| ....|....| ....|....|
TFL1.1   RVEVQGGDLR SFFTLVMTDP DVPGPSDPYL REHLHWYKTY PDHVYFA--- ----------
TFL1.2   RVEVQGGDLR SFFTLVMTDP DVPGPSDPYL REHLHWIVTD IPGTTDASF- ----------
TFL1.3   RVEVQGGDLR SFFTLVMTDP DVPGPSDPYL REHLHWIVTD IPGTTDASF- ----------
TFL1.5   RVEVQGGDLR SFFTLVMTDP DVPGPSDPYL REHLHWIVTD IPGTTDASFG EHIFSFIYPS
TFL1.4   RVEVQGGDLR SFFTLVMTDP DVPGPSDPYL REHLHWYEKT I--------- ----------
CEN      KVEIGGDDMR SSYALVMTDP DAPSPSDPCL REHLHWLVYK HLYTYCTEFG ----------

                 130        140        150        160        170        180
         ....|....| ....|....| ....|....| ....|....| ....|....| ....|....|
TFL1.1   ---------- ---------- -RREVVNYES PRPNIGIHRF VFVLFKQKKR NSVSSPGVRD
TFL1.2   ---------- ---------- -GREVVNYES PRPNIGIHRF VFVLFKQKKR NSVSSPGVRD
TFL1.3   ---------- ---------- -GREVVNYES PRPNIGIHRF VFVLFKQKKR NSVSSPGVRD
TFL1.5   STYMTQKDKH MHDIYDYSSF VGREVVNYES PRPYIGIHRF VFVLFKQKKR NSVSSPGVRD
TFL1.4   ---------- ---------- ---------- ---------- ---------- ----------
CEN      ---------- ------YMIF SGREVVSYEA PRPVIGIHRY VFTLFKQKSR NTARAPQSRD

                 190        200        210
         ....|....| ....|....| ....|....| ...
TFL1.1   RFNTRQFAEK NELGLPVAAV YFNAQRETAA RRR
TFL1.2   RFNTRQFAEK NELGLPVAAV YFNAQRETAA RRR
TFL1.3   RFNTRLFAEK NELGLPVAAV YFNAQRETAA RRR
TFL1.5   RFNTQRFAEK NELGLPVAAV YFNAQRETAA RRR
TFL1.4   ---------- ---------- ---------- -RS
CEN      CFNTRNFAVE NGLGLPVAAV FFNCQRETAC RRR
```

Figure S1: Amino acid sequence alignment of six representatives of the TFL-like clade in garlic genome.

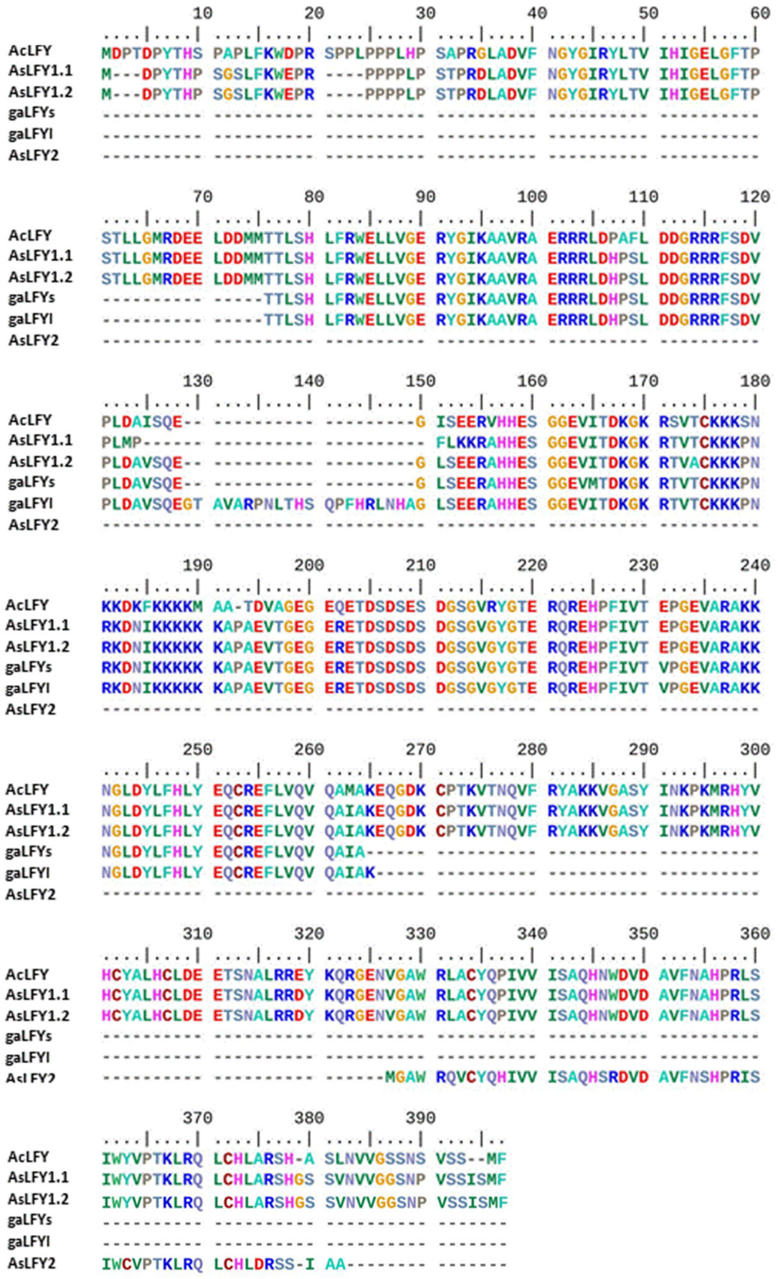

Figure S2: Amino acid sequence alignment of *LFY* homologs from garlic (As) and onion (Ac). Two spliced transcripts, previously reported as *gaLFYl* and *gaLFYs* [13], have high homology with *AsLFY1*.

Table S1: Homologs of the main known flowering-related genes discovered in garlic genome.

Table S2: Sequences of FT and LFY proteins of different species downloaded from the NCBI database (https://www.ncbi.nlm.nih.gov/, accessed on 15 February 2021).

Name	Accession
AcCEN	AOP17764.1
AcFT1	AGZ20207.1
AcFT2	AGZ20208.1
AcFT3	AGZ20209.1
AcFT4	AGZ20210.1
AcFT5	AGZ20211.1
AcFT6	AGZ20212.1
AcFT7	Asa7G06501.1
AcTFL1	AOP17765.1
AsCEN	Asa4G03276.1
AsFT1.1	Asa7G06383.1
AsFT1.2	Asa7G06386.1
AsFT10	Asa7G06404.1
AsFT11	Asa6G01542.1
AsFT2.1	Asa6G06199.1
AsFT2.2	Asa6G06200.1
AsFT3.1	Asa6G00732.1
AsFT3.2	Asa6G01063.1
AsFT4.1	Asa6G00187.1
AsFT4.2	Asa6G00188.1
AsFT4.3	Asa8G01025.1
AsFT4.4	Asa8G01036.1
AsFT5.1	Asa0G05138.1
AsFT5.2	Asa8G04470.1
AsFT6.1	Asa2G02821.1
AsFT6.2	Asa7G06501.1
AsFT8.1	Asa5G01472.1
AsFT8.2	Asa6G04367.1
AsFT9.1	Asa2G04443.1
AsFT9.2	Asa2G04445.1
AsTFL1.1	Asa5G02970.1
AsTFL1.2	Asa5G02971.1
AsTFL1.3	Asa5G02972.1
AsTFL1.4	Asa0G01615.1
AsTFL1.5	Asa4G04758.1
AtBFT	NP_201010.1
AtCEN	NP_180324.1
AtFT	NP_001320342.1
AtMFT	NP_173250.1
AtTFL1	NP_196004.1
AtTSF	NP_193770.1

BdFT	XP_003564300.1
BdFT	XP_003565602.1
BdFT	XP_003575305.1
BdFT	XP_003580019.1
BdFT	XP_003576940.1
BdFT	XP_010238354.1
BdFT	XP_003563772.1
BdFT	XP_003571147.1
BdFT	XP_003568088.1
BdFT	XP_003569807.1
BdMFT	XP_003566347.1
BdMFT	XP_003568618.1
BdMFT	XP_024312894.1
BdTFL1	XP_003575068.1
BdTFL1	XP_003579695.1
BdTFL1	XP_003578901.1
BdTSF	XP_010238588.1
BdTSF	XP_003578751.1
OsFT	XP_015640513.1
OsFT	XP_015641951.1
OsFT	XP_015642519.1
OsFT	XP_015611892.1
OsFT	XP_015627229.1
OsFT	XP_015633387.1
OsFT	XP_015617569.1
OsFT	XP_015611907.1
OsFT	XP_015641828.1
OsFT	XP_015624852.1
OsFT	XP_015630200.1
OsMFT	XP_015641734.1
OsMFT2	NP_001359127.1
OsTFL1	XP_015624118.1
OsTFL1	XP_015634144.1
OsTFL1	XP_015618522.1
OsTFL1	XP_025876956.1
OsTSF	XP_015619436.1
